# Strongly Basic Anion Exchange Resin Based on a Cross-Linked Polyacrylate for Simultaneous C.I. Acid Green 16, Zn(II), Cu(II), Ni(II) and Phenol Removal

**DOI:** 10.3390/molecules27072096

**Published:** 2022-03-24

**Authors:** Monika Wawrzkiewicz, Anna Wołowicz, Zbigniew Hubicki

**Affiliations:** Department of Inorganic Chemistry, Faculty of Chemistry, Institute of Chemical Sciences, Maria Curie-Sklodowska University in Lublin, M. Curie-Sklodowska Sq. 2, 20-031 Lublin, Poland; anna.wolowicz@mail.umcs.pl (A.W.); zbigniew.hubicki@mail.umcs.pl (Z.H.)

**Keywords:** heavy metals, C.I. Acid Green 16, phenol, anion exchange resin, adsorption, removal, column test

## Abstract

The adsorption ability of Lewatit S5528 (S5528) resin for C.I. Acid Green 16 (AG16), heavy metals (Zn(II), Cu(II) and Ni(II)) and phenol removal from single-component aqueous solutions is presented in this study to assess its suitability for wastewater treatment. Kinetic and equilibrium studies were carried out in order to determine adsorption capacities, taking into account phase contact time, adsorbates’ initial concentration, and auxiliary presence (NaCl, Na_2_SO_4_, anionic (SDS) and non-ionic (Triton X100) surfactants). The pseudo-second-order kinetic model described experimental data better than pseudo-first-order or intraparticle diffusion models. The adsorption of AG16 (538 mg/g), phenol (14.5 mg/g) and Cu(II) (5.8 mg/g) followed the Langmuir isotherm equation, while the uptake of Zn(II) (0.179 mg^1−1/n^L^1/n^/g) and Ni(II) (0.048 mg^1−1/n^L^1/n^/g) was better described by the Freundlich model. The auxiliary’s presence significantly reduced AG16 removal efficiency, whereas in the case of heavy metals the changes were negligible. The column studies proved the good adsorption ability of Lewatit S5528 towards AG16 and Zn(II). The desorption was the most effective for AG16 (>90% of dye was eluted using 1 mol/L HCl + 50% *v*/*v* MeOH and 1 mol/L NaCl + 50% *v*/*v* MeOH solutions).

## 1. Introduction

**Figure 1:**
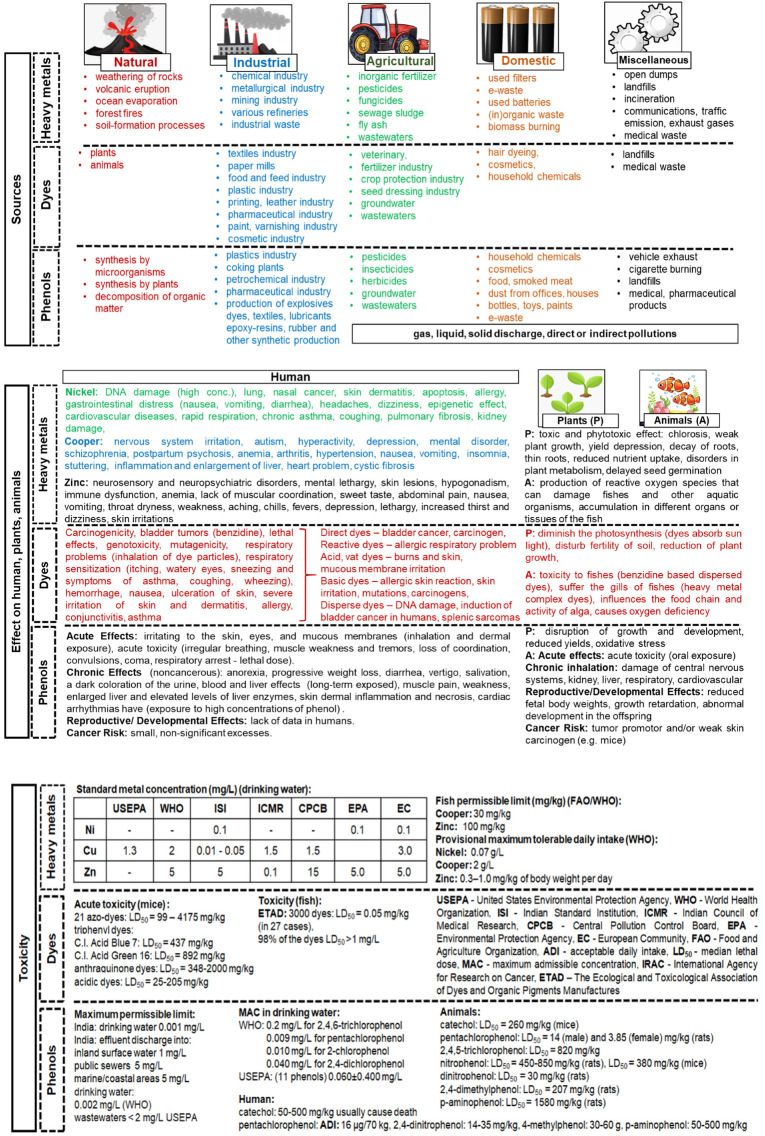
Sources of heavy metals, dyes and phenols in the environment and their impact on human, plants and animals and toxicity [8,15,16,17,18,19,20,21,22,23,25].

Nowadays, one of the main problems of global rank is the increasing amount of pollutants introduced into the environment as a result of rapid industrialization and urbanization processes [1]. Particularly dangerous are wastewaters discharged from different branches of industry into the environment containing dyes, heavy metals and phenol [2,3]. The main natural and anthropogenic sources of heavy metals [4,5], dyes [6,7] and phenolic compounds [8,9] in the environment, their effect on living organisms [10,11,12,13,14], as well as their toxicity [15,16,17,18,19,20,21,22,23,24,25] are presented in Figure 1. These wastewaters harm the aquatic ecosystem, humans and plants. Additionally, the components of wastewaters are toxic, non-biodegradable, mutagenic, and carcinogenic, and are able to accumulate in living organisms, resulting in serious health problems and even death [10]. Therefore, the removal of these pollutants from wastewater to protect the environment and human health is urgently required.

Various treatment processes are available for contaminant removal (chemical precipitation, ion exchange, adsorption, solvent extraction, membrane filtration, advanced oxidation, reverse osmosis, etc.). Among them, adsorption is one of the most popular and recognized techniques [10,26,27,28]. The undoubted advantages of adsorption include ease of operation, and an excellent ability to remove the contaminants present in wastewaters.

Even at low concentrations, a wide range of adsorbents are available; moreover, adsorbents can be regenerated and reused, and have low sludge generation [27,28].

Among various adsorbents applied for heavy metals, dye and phenol removal, functionalized polymeric adsorbents have been recognized as a practical alternative to activated carbon due to the possibility of high (in)organic contaminate removal efficiency, simple and nondestructive regeneration, as well as a favorable adsorption mechanism [28]. The uptake of heavy metals, dyes and phenol could be improved by applying the appropriately selected ion exchange resins. As was found by Lee et al. [29], the uncharged polymeric resins showed a lower capacity for phenol compared to activated carbon; therefore, the presence of the functional groups also play a significant role in removal efficiency. The use of the anion exchange resins could improve phenol uptake, whereas pH is one of the main parameters that effects phenol removal ability. Depending on pH, phenols can bind via the ion exchange or adsorption mechanism [30]. The application of the strongly basic anion exchanger Purolite A510 (the chloride form) for phenol removal shows that uptake depends on the solution’s pH, and increases sharply with increasing pH. This fact results from the higher dissociation ability of the phenol at a higher pH than its pK_a_ (9.83). It has been considered that the uptake of a phenol occurs only at the active adsorption sites of the anion exchangers by adsorption or ion exchange (distinguishing between molecular adsorption or ion exchange depending on the pH was not indicated). Moreover, not only the solution’s pH but also the form of the functional groups of ion exchange resins influence the exchange of phenolate in the resin phase [31]. Studies on phenol removal by using the Amberlite IRA420 proved that their retention occurs by adsorption at acidic pH and by both adsorption and ion exchange at an alkaline pH. Phenol removal efficiency was constant at a pH below 8, whereas this increased at an alkaline pH from 9 to 14 [32]. Additionally, the separation of the mixtures of alkylphenols by ion exchange resins showed the selective adsorption of 2,6-xylenol from these mixtures. The separation is governed by the acid–base interactions between phenolic OH^−^ groups and the functional groups of the resin. The separation was favored by the thermodynamic selectivity with diffusional selectivity [33].

Similar to phenol removal, a much higher adsorption ability of resin containing functional groups compared to uncharged polymers was found for dyes [34,35]. The adsorption capacities, *q_e_* obtained during C.I. Basic Blue 3 dye removal using Lewatit MonoPlus SP 112 (cation exchange resin) was much higher (560.7–637.6 mg/g, temp. 293–323 K) than using Amberlite XAD7 (28.9–66.5 mg/g) and Dowex Optipore SD2 (270.9–247.5 mg/g) copolymers. Similar observations were made during C.I. Direct Yellow 50 sorption on Amberlite IRA478, Amberlite IRA958, Lewatit MonoPlus MP68, Amberlite IRA900 (*q_e_* = 354.8–534.8 mg/g), Lewatit VPOC1064 (*q_e_* = 19.4 mg/g), and Amberlite XAD7 (*q_e_* = 27.9 mg/g). The high adsorption ability was proven during the application of anion exchange resins towards various dyes, e.g., C.I. Reactive Red 120, C.I. Reactive Red 198, C.I. Reactive Black 5 on S6328A (strongly basic anion exchange resin, SBA), and MP62 (weakly basic anion exchange resin, WBA) [36]. More examples are related to the removal of tartrazine on Amberlite FPA51 (WBA, *q_e_* = 140.8 mg/g) [37], Sunset Yellow on Amberlite FPA51 (WBA, *q_e_* = 130.6 mg/g) [38], C.I. Direct Red 75 on Amberlite IRA67 (WBA, *q_e_* = 994.9 mg/g), and Amberlite IRA458 (SBA, *q_e_* = 430.8 mg/g) [39]. Investigating the adsorption of C.I. Acid Orange 7 (AO7), C.I. Reactive Black 5 (RB5), and C.I. Direct Blue 71 (DB71) dyes on the weakly (Amberlite IRA67, Lewatit MonoPlus MP62, Amberlyst A23), intermediate (Lewatit MonoPlus MP64 and Amberlite IRA478RF) and strongly (Amberlite IRA458, Amberlite IRA958, Amberlite IRA900, Amberlite IRA910, Lewatit MonoPlus MP500, Lewatit MonoPlus M500 and Lewatit MonoPlus M600) basic anion exchangers, it was concluded that Amberlite IRA958 showed the best adsorption performance towards dyes (*q_e_* = 1370.4 mg/g for AO7, *q_e_* = 1655.2 mg/g for RB5, and *q_e_* = 1630.6 mg/g for DB71 [40,41,42,43]).

Many examples of heavy metal ion removal using various ion exchangers can be found in the literature [44,45,46,47,48,49,50]. Cu(II), Ni(II), Co(II), Zn(II), Pb(II) ions were effectively removed by the strongly (Lewatit MonoPlus M500, Lewatit MonoPlus MP 500 [45], Amberlite IRA458, Amberlite IRA958 [46,47], Amberlite IRA 402 [47], Amberlite IRA402 [48]) and weakly basic (Amberlite IRA67 [46]) anion exchange resins in the presence of various complexing agents. Our previous studies concerning Co(II), Ni(II), Cu(II) and Zn(II) removal on the weakly (Purolite A830, Lewatit MonoPlusTP220), intermediate (Amberlite IRA478RF) and strongly (Dowex MSA1, Dowex MSA2, Lewatit MonoPlus SR7, Purolite A400TL) basic anion exchangers, as well as the chelating (Purolite S984) and carbon-based (Lewatit AF5) adsorbent showed that the highest capacity was obtained for the resin of bis-picolyamine functional groups [49,50,51,52,53,54,55].

Despite the many examples of heavy metals, dyes and phenol removal on anion exchange resins available in the literature, there are no examples of comprehensive studies using a strongly basic anion exchanger to remove these three types of contaminant. In addition, the use of Lewatit S5528 to remove inorganic as well as organic contaminants has not yet been discussed. Therefore, the aim of the presented research (novelty of the work) was to evaluate the adsorption properties of the strongly basic anion exchange resin Lewatit S5528 towards heavy metal ions (Cu(II), Zn(II), Ni(II)), C.I. Acid Green 16 dye and phenol in single-component solutions, as well as to establish the possible interactions between resin and adsorbates. The impacts of phase contact time, adsorbate initial concentration, and auxiliary presence (NaCl, Na_2_SO_4_, sodium dodecyl sulfate (SDS), 2-[4-(2,4,4-trimethylpentan-2-yl)phenoxy]ethanol (Triton X100) were evaluated on adsorbate’s sorption effectiveness on Lewatit S5528 resin. Kinetic, equilibrium, desorption and reuse possibility were also discussed.

## 2. Results

### 2.1. Isotherm Equilibrium Studies

Adsorption isotherm models are very useful for explaining the interaction between an adsorbate and adsorbent in a given system at equilibrium. They describe the affinity of the toxic substance towards the adsorbent. This can be completed by examining the relationship between the concentration of adsorbate at equilibrium in the liquid phase and the solid phase at a specific temperature. The data obtained allow the evaluation of adsorption as a process of physical or chemical nature, too. Moreover, the calculation of the sorption capacity as a value determining the maximum retention of adsorbate is extremely important from a practical point of view. It allows us to obtain information about the application of a given adsorptive material in industrial technologies of pollutant removal by adsorption techniques. The amounts of AG16, phenol and heavy metal ions sorbed by S5528 at equilibrium (*q_e_*), known as the adsorption capacities, were calculated from Equation (1):

(1) $$q_{e}=\frac{\left(C_{0}-C_{e}\right)}{m}\;V$$

where *C*_0_ and *C_e_* (mg/L) are adsorbate concentration in the solution before adsorption and at equilibrium, respectively; *V* (L) is the volume of the adsorbate solution; and *m* (g) is the mass of Lewatit S5528.

In this study, three popular isotherm models such as Langmuir, Freundlich and Temkin were applied for the description of dye, phenol and heavy metal ion uptake by anion exchange resin at equilibrium. They can be presented using the following Equations [56,57]:

(2) $$q_{e}=\frac{k_{L}Q_{0}C_{e}}{1+C_{e}k_{L}}$$

(3) $$q_{e}=k_{F}C_{e}^{1/2}$$

(4) $$q_{e}=\frac{RT}{b_{T}}lnAC_{e}$$

where *k_L_* (L/mg) is the Langmuir constant parameter of adsorption equilibrium; *Q*_0_ (mg/g) is the monolayer adsorption capacity;$\;k_{F}$(mg^1−1/n^ L^1/n^/g) and *n* are Freundlich constants related to adsorption capability and adsorption intensity, respectively; *b_T_* (J g/mol mg) is the Temkin constant related to the heat of adsorption; *A* (L/mg) is the Temkin isotherm equilibrium binding constant; *R* is the gas constant (8.314 J/mol K); and *T* (K) is temperature.

The Langmuir isotherm (Equation (2)) is the basic isotherm of adsorption. It assumes that an adsorbate can form a so-called monolayer of molecules on the surface of an adsorbent, interacting with adsorption sites and not interacting with each other. The adsorbed molecules are characterized by a certain desorption probability. Both probabilities depend on the temperature and the adsorption energy. The Freundlich isotherm (Equation (3)) describes the adsorption on heterogeneous (energetically heterogeneous) surfaces. If the molecules in the adsorption layer have a certain mobility at the surface, the adsorption centers with the highest adsorption energy will be saturated first. Centers with increasingly lower energies are then saturated. The variation of adsorption heat may also be caused by interactions between adsorbed molecules. The Temkin isotherm (Equation (4)) describes adsorption on a heterogeneous solid. This isotherm corresponds to a continuous, infinite (unlimited by minimum or maximum energy) energy distribution of the adsorption sites. Temkin’s equation assumes that the heat of adsorption of all molecules in the layer decreases linearly due to the adsorbent–adsorbate interaction, and adsorption is characterized by the uniform distribution of binding energy.

The parameters characteristic for selected isotherm models as well as Marquardt’s percent standard deviation (*MPSD*), determination coefficient (*R*^2^) and adjusted R-squared ($R_{adj}^{2}$) [58], which are essential for the evaluation of the best fitting model using linear (L) and non-linear regression (NL), are presented in Table 1.

**Figure 2:**
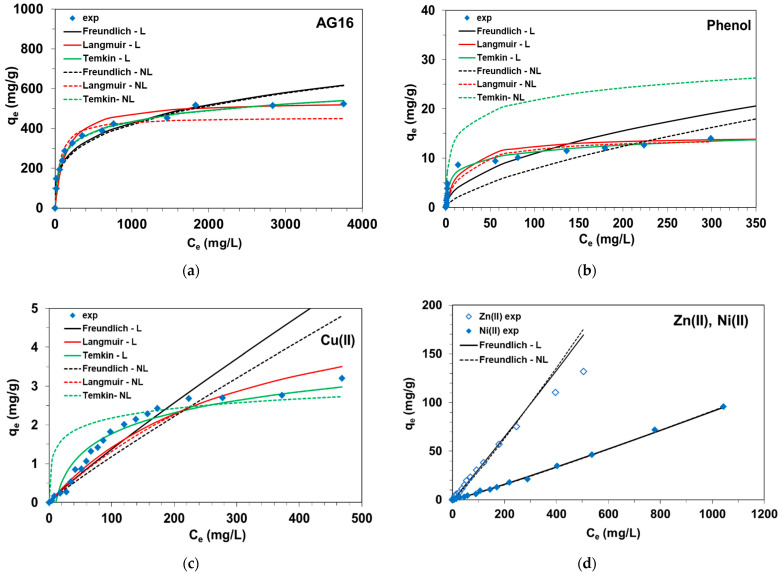
Isotherm experimental data of (**a**) AG16, (**b**) phenol, (**c**) Cu(II), (**d**) Zn(II) and (Ni) on S5528 anion exchange resin corresponding with fitting curves to the Freundlich, Langmuir and Temkin isotherm equations using linear (L) and non-linear (NL) regression.

Figure 2 shows the fitting curves of the isotherm models to the experimental data.

Considering AG16 and phenol adsorption on the anion exchanger, it can be seen that the Langmuir model provided the better description of the experimental data, rather than the Freundlich or Temkin models. The values of the determination coefficients *R^2^* equaled 0.997 and 0.914 for AG16, and 0.989 and 0.943 for phenols using linear and non-linear regression, respectively. These values were higher than those determined for the Freundlich (*R*^2^ = 0.928–0.949 for AG16, *R*^2^ = 0.780–0.861 for phenol) or Temkin (*R*^2^ = 0.984–0.986 for AG16, *R*^2^ = 0.978–0.969 for phenol) models. Similar changes of the values of adjusted *R*^2^ were observed. *MPSD* values were the smallest for the Langmuir model compared with the Freundlich or Temkin models. Linear regression appeared to be more appropriate than non-linear regression in describing the AG16-S5528 (Figure 2a) and phenol-S5528 (Figure 2b) adsorption systems. The distribution of experimental points with an apparent plateau in the case of the AG16 and phenol adsorption is also consistent with the assumptions of the Langmuir model. The monolayer adsorption capacities (*Q*_0_) determined from the Langmuir model were 538 mg/g for AG16 and 14.5 mg/g for phenol and correlated with the experimental values (524 mg/g and 14.3 mg/g for AG16 and phenol, respectively). The values of the dimensionless equilibrium parameter *R_L_* [59] (determined as $R_{L}=\frac{1}{1+k_{L}C_{0}}$), being essential characteristics of the Langmuir model, were calculated too. The *R_L_* values ranged from 0.059 to 0.007 for AG16 (*C*_0_ of dye between 1000 and 9000 mg/L) and from 0.956 to 0.042 for phenol (*C*_0_ of phenol between 5 and 500 mg/L) under the studied conditions. The adsorption of AG16 and phenol onto S5528 is favorable, as *R_L_* values were found to be in the 0–1 range.

The analysis of the distribution of experimental points in the case of Cu(II) adsorption on anion exchanger did not allow an unambiguous assessment of which of the isotherm models used was the most appropriate to describe these data (Figure 2c). In the equilibrium concentration (*C_e_*) range from 5 to 173 mg/L of Cu(II), an almost proportional increase in the *q_e_* values were observed, which would suggest that the Freundlich adsorption model described the experimental data well in the mentioned concentration range. The Freundlich isotherm constant, *1/n*, gives an idea for the favorability of the adsorption process. The 1/n value calculated for Cu(II) adsorption on S5528 was less than 1, which indicated favorable adsorption conditions. Thereafter, it was observed that the changes in *q_e_* were not so significant. Analysis of the Langmuir isotherm parameters determined by non-linear regression confirmed by higher *R*^2^ and $R_{adj}^{2}$ values and lower *MPSD* compared to other models would suggest its applicability to describe experimental data in the higher range of *C_e_* values (*Q*_0_ = 8.3 mg/g). 

The equilibrium adsorption data of Zn(II) and Ni(II) on S5528 showed a simple linear relationship between *C_e_* and *q_e_*, as presented in Figure 2d. Fitting of the experimental data is only possible to the linear and non-linear forms of the Freundlich isotherm. The *k_F_* values calculated for Zn(II) and Ni(II) by non-linear regression equaled 0.179 and 0.048 mg^1−1/n^ L^1/n^/g, respectively. 1/*n* values calculated using the linear and non-linear regression were found to be close to 1 (e.g., 1.11 for Zn(II) and 1.09 for Ni(II) by non-linear regression). The *R*^2^ ($\mathrm{and}\;R_{adj}^{2})\;$values of the Freundlich model were obtained as 0.984 (0.981) for the Zn(II)-S5528 system and 0.999 (0.999) for the Ni(II)-S5528 system.

Evaluation of the adsorption of toxic substances from a multicomponent mixture would be interesting, but difficult to assess reliably, as the oxidation of the dyes or their degradation may occur in such systems. It can also be concluded that the sorption capacity, with respect to the individual adsorbates removed from the mixture, may differ from that determined in single-component solutions.

Table 2 shows a comparison of the sorption properties of the polyacrylate anion exchanger S5528 against other sorbents used to remove AG16, phenol and heavy metal ions based on a literature review.

The retention of dye and phenol by the anion exchanger occurs due to the attraction between the anionic form of AG16 and phenol under experimental conditions and the quaternary ammonium functional groups of positive charge of the resin. The π–π interactions between aromatic rings present in the dye and phenol and resin matrix, as well as hydrogen bonds, can occur too. The adsorption of large organic species by S5528 resin is favored not only by the hydrophilic nature of the resin matrix but also as a result of the possibility of van der Waals interactions forming between the aliphatic part of the matrix and the adsorbates [68,69,70,71]. Different heavy metal adsorption efficiency is correlated with their forms, in which they exist in the dilute and more concentrated acidic solutions. Copper(II) exists in HCl solutions as Cu^2+^, CuCl^+^, CuCl_2_ and CuCl_3_^−^, CuCl_4_^2−^; zinc(II) as Zn^2+^, ZnCl^+^, ZnCl_2_ and ZnCl_3_^−^, ZnCl_4_^2-^; and nickel(II) as Ni^2+^, NiCl^+^, and NiCl_2._ Their ionic radii are: 69 pm for Ni(II), 73 pm for Cu(II) and 74 pm for Zn(II). An increase in the anionic forms of heavy metals in the solution increases the adsorption ability on the anion exchange resin; therefore, the removal efficiency is the highest for Zn(II) (fraction of anionic zinc chloro-complexes is higher compared to copper(II) ones) but in the concentrated HCl solution the competition effect can be responsible for the slight removal of efficiency reduction [72]. The mechanism of heavy metal ions adsorption onto the anion exchanger can be described as ion-exchange, which is illustrated in [55].

### 2.2. Kinetic Studies

It is well known that predicting the adsorption kinetics of contaminants is important for the design of adsorption systems. The speed of contaminant removal depends on both the physicochemical properties of the adsorbent and the conditions in the system. The phase contact time is an important parameter influencing effectiveness of uptake of adsorbate, such as heavy metal ions (i.e., Cu(II), Zn(II), Ni(II)), AG16 dye and phenol. The kinetic experiments in the systems under discussion were conducted to evaluate the kinetic parameters. The amount of heavy metals, dye or phenol sorbed by the strongly basic anion exchanger Lewatit S5528 at time *t* (*q_t_*), as well as the percentage removal (%*R*) of adsorbate, were calculated from the Equations (5) and (6):

(5) $$q_{t}=\frac{\left(C_{0}-C_{t}\right)}{m}\;V$$

(6) $$\%R=\frac{\left(C_{0}-C_{t}\right)}{C_{0}}100\%$$

where *C*_0_ and *C_t_* (mg/L) are the adsorbate concentration in the solution before and after sorption time *t*, respectively; *V* (L) is volume of the adsorbate solution; and *m* (g) is mass of Lewatit S5528.

**Figure 3:**
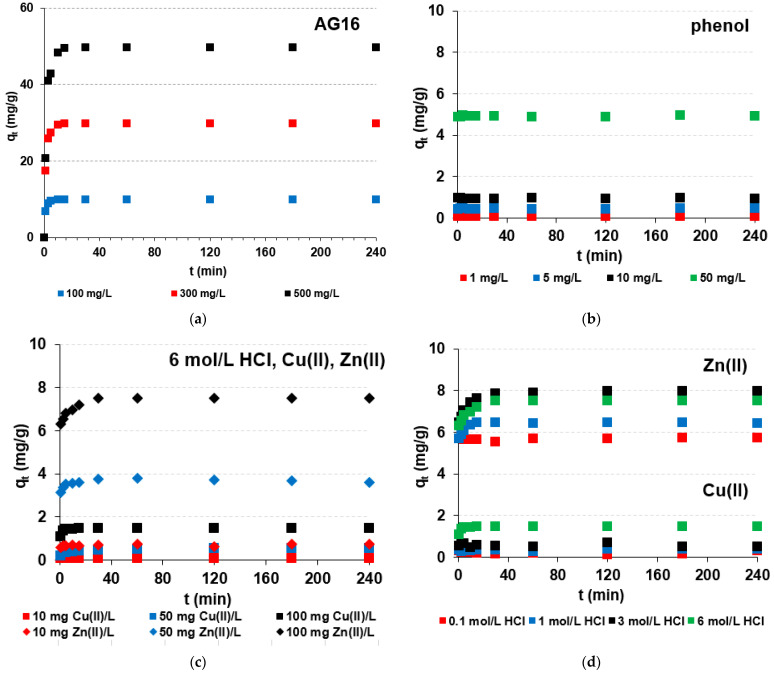
Kinetic plot in the adsorbate-S5528 systems. Impact of (**a–d**) phase contact time, initial concentrations of (**a**) AG16, (**b**) phenol and (**c**) metal ions, as well as (**d**) HCl concentration on Zn(II) and Cu(II) removal (*C*_0_ = 100 mg/L).

The effects of phase contact time (1–240 min), initial concentration of dye (100, 300, 500 mg/L), heavy metals (10, 50, 100 mg/L) and phenol (1, 5, 10, 50 mg/L), as well as acid concentrations in the case of heavy metals (0.1–6 mol/L HCl, 0.1–0.9 mol/L HCl–0.9–0.1 mol/L HNO_3_ systems), are presented in Figure 3 (chosen examples).

During the AG16 adsorption on S5528, the amount of dye adsorbed increased with the phase contact time increase (e.g., for 1 min *q_t_* = 20.7 mg/L, whereas for 15 min *q_t_* = 49.5 mg/L, 500 mg/L) as well as with the initial dye concentration increase (*q_t_* = 6.8 mg/L, 100 mg/L; *q_t_* = 17.4 mg/L, 300 mg/L; *q_t_* = 20.7 mg/L, 500 mg/L for 1 min). This fact may be explained due to the high driving force for mass transfer at a high initial dye concentration [73]. The time required to reach the equilibrium in the AG16-S5528 system was found to be 10, 15 and 30 min in the solutions of the initial dye concentrations 100, 300 and 500 mg/L, respectively. There are two steps in adsorption: (i) rapid adsorption at the beginning (1–30 min), where *q_t_* values increase rapidly (kinetic curves have a steep shape); followed by (ii) the second (30–240 min) much slower adsorption due to the saturation of adsorption sites on the adsorbent surface, where *q_t_* values increase slightly or not at all (plateau on kinetic curves is observed) (Figure 3a). The adsorption capacities were 9.9 mg/g, 29.9 mg/g and 49.7 mg/g for a system of 100, 300 and 500 mg AG16/L, whereas the removal efficiency after 10 min of phase contact time were 98.8%, 97.4% and 96.5%; then, at 240 min, these values were close to 99–100% in all cases.

For phenol uptake, the *q_t_* possessed similar values for 1–240 min of phase contact time and were in the ranges of 0.07–0.09 mg/g, 0.42–0.48 mg/g, 0.93–0.97 mg/g and 4.87–4.96 mg/g for systems containing 1, 5, 10 and 50 mg/L of phenol, respectively. The adsorption capacities increased from 0.09 mg/g to 0.48 mg/g, 0.97 mg/g and to 4.7 mg/g with the phenol initial concentration increase from 1 to 50 mg/L (Figure 3b), and the removal efficiency was from 74.5% to 99.1%.

The adsorption of heavy metals (M(II)) on S5528 showed that the adsorption capacities increased with the initial concentration increase, e.g., for Cu(II) (Ni(II)) this value increased from 0.08 (0.73) mg/g to 0.55 (3.6) mg/g to 1.5 (7.5) mg/g for 10, 50 and 100 mg M(II)/L with the 6 mol/L HCl system (Figure 3c). With the increase in HCl concentration, the amount of heavy metals adsorbed on S5528 increased for Cu(II) and Zn(II) (Figure 3d) or stayed at a similar level (4–4.7 mg/g) for Ni(II). In the HCl-HNO_3_ systems, the *q_t_ (%R)* values were 0.13–1.17 mg/g (1–12%) for Cu(II), 5.5–6.4 mg/g (55–64%) for Zn(II), and 4.3–4.7 mg/g (42–47%) for Ni(II). Only for Zn(II), the values of *%R* and *q_e_* slightly increased with the increasing content of HCl in the mixture. The highest values of *%R* and *q_e_* equaled to 64% and 6.4 mg/g, respectively, were found in the 0.9 mol/L HCl–0.1 mol/L HNO_3_ solution. No meaningful impact of the increasing phase contact time on heavy metal ion uptake was observed. For example, the percentage removal of Ni(II) was found to be 44.3% after 1 min and 45.9% after 240 min of sorption in the 0.5 mol/L HCl–0.5 mol/L HNO_3_. The S5528 adsorption ability series in the HCl medium and HCl-HNO_3_ (i.e., 0.1 mol/L HCl–0.9 mol/L HNO_3_, 0.2 mol/L HCl–0.8 mol/L HNO_3_, 0.5 mol/L HCl–0.5 mol/L HNO_3_, 0.8 mol/L HCl–0.2 mol/L HNO_3_, 0.9 mol/L HCl–0.1 mol/L HNO_3_) systems were Zn(II) > Ni(II) > Cu(II).

The pseudo-first-order kinetic model (PFO, Equation (7)), the pseudo-second-order kinetic model (PSO, Equation (8)) as well as the intraparticle diffusion model (IPD, Equation (9)) were applied for AG16, Cu(II) and Zn(II) kinetics data modelling using the following formulas:

(7) $$q_{t}=q_{e}\left(1-e^{-k_{1}t}\right)$$

(8) $$q_{t}=\frac{k_{2}q_{e}^{2}}{1+k_{2}q_{e}t}$$

(9) $$q_{t}=k_{i}t^{0.5}$$

where *q_e_* and *q_t_* (mg/g) are adsorbate amounts sorbed at the equilibrium and at any time *t*; *k*_1_ (1/min) and *k*_2_ (g/mg min) are rate constants of sorption determined from PFO and PSO equations, respectively; and *k_i_* (mg/g min^0.5^) is intraparticle diffusion rate constant [74,75,76].

Due to the kinetic curve shape and position of experimental point, the determination of the kinetic parameters was not possible in the cases of phenol and Ni(II). Table 3 presents the collected kinetic parameters using the PFO, PSO and IPD models with the linear (L) and non-linear (NL) regression for selected adsorbates.

**Figure 4:**
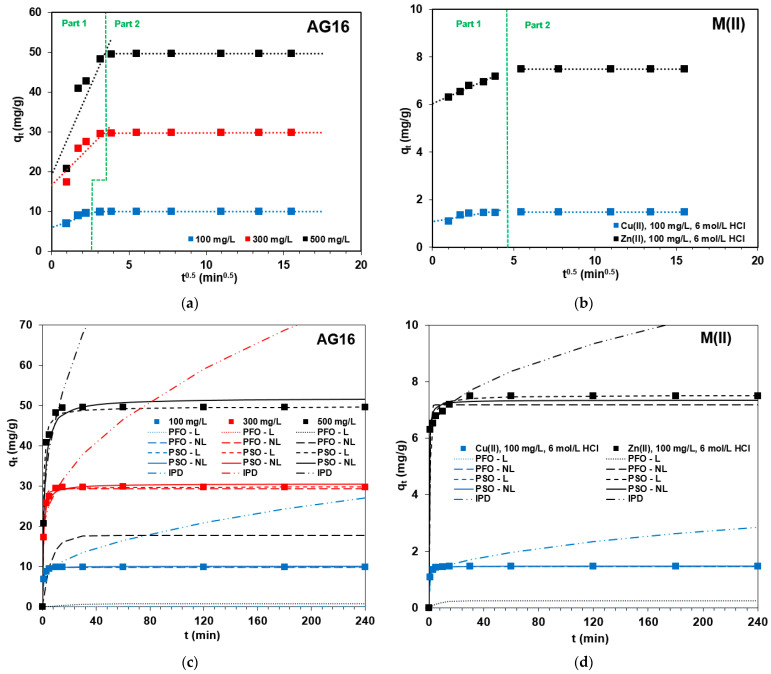
Intraparticle diffusion model applied for adsorption of (**a**) AG16 and (**b**) heavy metal ions as well as the fitting of the experimental data obtained for (**c**) AG16 and (**d**) heavy metals to the PFO, PSO and IPD models.

The Lagergren equation is not suitable to describe the AG16 and heavy metal sorption kinetics on anion exchange resin S5528 due to the small values of the determination coefficients being in the range 0.239–0.435 for AG16 and 0.524–0.788 for heavy metals, as well as due to high inconsistency of the adsorption capacity obtained experimentally and calculated from the PFO model with the L regression. Similar observations were also found for the PFO model with the NL regression. In this case, the *R*^2^ values were higher compared to PFO-L (0.829–0.961 for AG16, 0.829–0.953 for heavy metals) but the values of adsorption capacities varied considerably. The $R_{adj}^{2}$ values were in the range from 0.780 to 0.949 for AG16, Cu(II) and Zn(II), and are usually smaller compared to the PSO-NL model; however, the *MPSD* values (0.0071–5.0626) were higher, and therefore the PFO-NL model was excluded as the best one to describe the adsorption of AG16, Cu(II) and Zn(II) on S5528. Much higher determination coefficients were obtained for the PSO model both with L (1.000) and NL (0.766–0.973) regression. Moreover, the error analysis proved that this model found applicability due to the smallest values of *MPSD* (0.0026–0.0309), as well as due to a high agreement between the calculated and experimentally obtained values of the sorption capacity (e.g., *q_e_* = 9.9 mg/g and *q_e,exp_* = 9.9 mg/g for AG16). The IPD model describes the adsorption process if the plot *q_t_* versus *t*^0.5^ gives a straight line. Generally, the kinetic curves illustrate multi-linearity, and the adsorption data can be fitted with two or three straight lines [76]. In the initial step, the external surface adsorption or instantaneous adsorption occurs; in the second one, the intraparticle diffusion plays an important role; in the third part, the equilibrium is approached (adsorption slows when surface coverage is nearly complete) [77]. Two separate stages of adsorption can be distinguished in Figure 4a,b, both for AG16 as well as heavy metals.

Despite the quite high determination coefficient (0.676–0.977), the intercept of the lines do not pass through the origin, indicating that the intraparticle diffusion (diffusion into the polymer network) is not only the rate controlling step. The value of the intercept indicates the thickness of the boundary layer. Moreover, its positive value indicates that there is rapid adsorption in short time, whereas the multilinearity indicates that multiple mechanisms control the process [76,77]. Thus, it is believed that surface adsorption as well as intraparticle diffusion may occur simultaneously in this system. Literature data [62,78,79] has played a significant role in the intraparticle diffusion in the adsorption of AG16 by anion exchanger (Lewatit S6368), smectite clay, or biodegradable graft copolymer derived from amylopectin and poly(acrylic acid). Furthermore, an evaluation of the applicability of kinetic models in the adsorption of heavy metals, dyes, and phenol on anion exchangers and other adsorbents indicates that the PSO model is usually chosen as the best one to describe the removal of these pollutants [77,78,79,80]. This is consistent with our results, which are confirmed by the fitting plots shown in Figure 4c,d.

### 2.3. Effect of Auxiliary Presence on Adsorption Effectiveness

**Figure 5:**
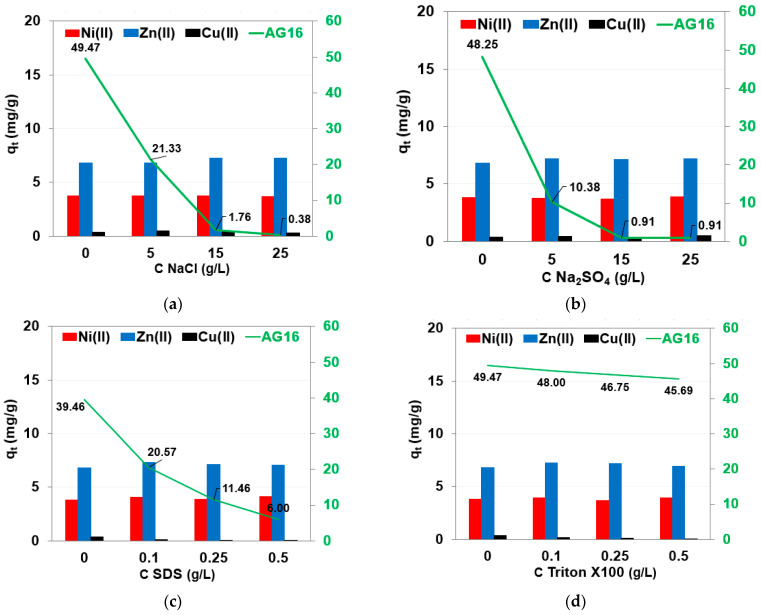
Effect of (**a**) NaCl, (**b**) Na_2_SO_4_, (**c**) SDS and (**d**) Triton X100 on adsorption efficiency of AG16, Ni(II), Zn(II), and Cu(II) on S5528 (*t* = 15 min, AG16 *C*_0_ = 500 mg/L; heavy metals *C*_0_ = 100 mg/L).

In the real wastewater of textile and metallurgical industries, in addition to dyes and heavy metals, auxiliary substances such as acids, bases, salts, surfactants, oxidizers, reducing agents, etc., may be present. Therefore, it is very important to study the effect of these auxiliaries on the removal efficiency of AG16 and heavy metals. The effects of salts (0–25 g/L NaCl, Na_2_SO_4_) and surfactants (0–0.5 g/L, the anionic surfactant (SDS) and nonionic surfactant Triton X100) on the adsorption of AG16 (500 mg/L) and heavy metal ions (100 mg/L) on the S5528 anion exchanger after 15 min of phase contact time were determined (Figure 5).

The effect of NaCl on heavy metal uptake was not significant; the amount of heavy metal ions remained at a similar level and *q_t_* values were close to ≈0.5 mg/g for Cu(II), 4 mg/g for Ni(II) and 7 mg/g for Zn(II), and did not change significantly with the increasing amount of added NaCl. Similar changes of *q_t_* values were observed with Na_2_SO_4_ in the M(II)-S5528 systems. In the case of AG16, the presence of NaCl as well as Na_2_SO_4_ drastically reduced *q_t_* values from 49.7 mg/g to 0.38 mg/g (NaCl), or from 48.3 to 0.91 mg/g (Na_2_SO_4_). The competitive sorption of the chloride and sulfate ions with the anionic form of the dye was observed. It was also observed during AG16 retention by the Lewatit S6368A [62].

SDS as the amphiphilic surfactant is ionized (Na^+^ is soluble in the aqueous phase, -OSO_3_C_12_H_25_ ions as the hydrophobic tail extends out of the water surface) [81]. The addition of SDS to the aqueous solution causes an increase in conductivity [62]. In solutions containing anionic dyes and the anionic surfactant SDS, electrostatic interaction (repulsion effect) between species with the same charge occurs and the removal efficiency decreases. As shown in Figure 5c, the reduction in AG16 removal efficiency after the addition of SDS surfactant was much greater than that for heavy metal ions, indicating that, in this case, the competence sorption was much more prominent. The introduction of divalent metal ions into the solution resulted in the formation of surfactant–metal ion interactions. The electrostatic interactions resulted in increasing micelle stability and the dodecyl sulfonic acid could be formed. Due to the nonionic Triton X100 addition to the AG16 system, the amount of AG16 adsorbed on S5528 reduction was observed (of 8%), but this reduction was smaller compared to SDS. In the case of heavy metal ions, Triton X100 addition did not significantly influence the adsorption efficiency. As was pointed out by Snukiškis et al. [82] Zn(II) adsorption in the presence of nonionic surfactant on Purolite C 106 exchanger proceeds as free cations on the basis of complex (ionic coordinate) bonding, free cations on the basis exclusively of coordinate bonding, and cations bonding to the surfactant molecules.

### 2.4. Desorption and Reuse Studies

Regeneration of the adsorbent and its subsequent use are the main factors determining its operating costs. Regeneration is necessary to restore the original capacity of the adsorbent for reuse. The heavy metal ions and dye desorption rate (*% D*) can be calculated from the Equation (10):

(10) $$D=\frac{m_{des}}{m_{ads}}100\%$$

where *m_des_* (mg) is mass of adsorbate eluted from the resin, and *m_ads_* (mg) is mass of adsorbate retained by the resin.

**Figure 6:**
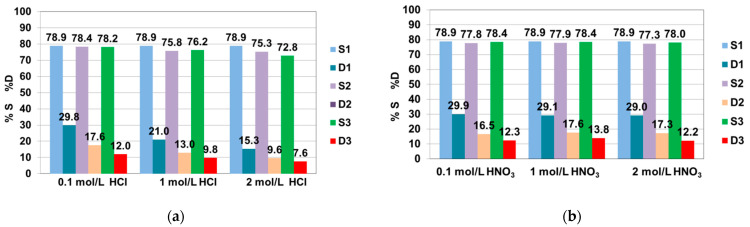

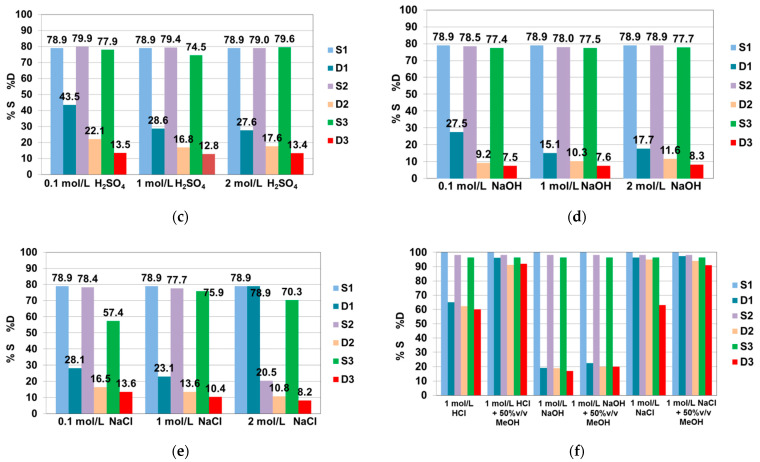
Regeneration of S5528 resin in three cycles of sorption (%S) and desorption (%D) for (**a**–**e**) Zn(II) and (**f**) AG16 dye using different eluting agents.

Desorption experiments were performed for Zn(II), Ni(II) (as their uptake during sorption tests were favorable compared with Cu(II) and phenol) and AG16. The desorption of Zn(II) and Ni(II) from S5528 resin was carried out using HCl, HNO_3_, H_2_SO_4_, NaOH and NaCl solutions of the concentrations 0.1, 1 and 2 mol/L. AG16 removal from the anion exchanger phase was investigated using 1 mol/L HCl, NaOH and NaCl aqueous methanol (50% *v*/*v*) solutions. The studies were performed in three cycles of sorption (initial concentration of Zn(II), Ni(II) and AG16 in each step of sorption was 100 mg/L) and desorption. The adsorption of Zn(II), Ni(II) and AG16 in three cycles equaled to 78.9–70.3% for Zn(II), 46.2–35.9% for Ni(II) and 100–96.3% for AG16. The desorption of Zn(II) from the S5528 phase was found to be 43.5% at the first cycle using 0.1 M H_2_SO_4_, which decreased to 13.5% at third cycle (Figure 6a–e). The highest desorption efficiency was obtained using 0.1 mol/L solutions as eluents, while the lowest regeneration efficiency was obtained using 2 mol/L solutions. Ni(II) desorption in the first cycle did not exceed 3.2–2.2%, using 0.1 mol/L solutions of HCl, HNO_3_, NaCl and H_2_SO_4_, respectively. 

Satisfactory adsorption and desorption were obtained in three cycles for AG16 dye. It could be seen that the 1 mol/L aqueous solutions of HCl, NaOH and NaCl showed lower regeneration efficiency compared to their counterparts in 50% *v*/*v* methanol medium. This was particularly evident in the case of the 1 mol/L HCl + 50% *v*/*v* MeOH and 1 mol/L NaCl + 50% *v/v* MeOH solutions, as presented in Figure 5f. More than 90% of AG16 could be eluted from the S5528 phase in the third cycle of desorption. These results confirm that the mechanism of AG16 uptake is of mixed nature and occurs due to chemical as well as physical interactions.

### 2.5. Column Studies

Column tests are often used to simulate the flow of wastewater containing toxic substances and to allow the practical application of the adsorbent to be assessed in wastewater treatment plants. To check the flow characteristics of the column, the relationship of the normalized concentration (*C/C*_0_) versus effluent volume (*V*) or time (*t*) is plotted. This is known as the breakthrough curve, and enables the calculation of the breakthrough (working) capacity (*C_w_*), as well as the weight (*D_w_*) and bed (*D_b_*) distribution coefficients from the following Equations (11)–(13) [34]:

(11) $$C_{w}=\frac{V_{bt}C_{0}}{v}$$

(12) $$D_{w}=\frac{\left(U-U_{0}-V_{v}\right)}{m}$$

(13) $$D_{b}=\frac{\left(U-U_{0}-V_{v}\right)}{v}$$

where *V_bt_* (mL) is column breakthrough volume; *C*_0_ (mg/L) is the influent concentration; *v* (mL) is resin volume in the column; *U* (mL) is volume of the effluent at C/C_0_ = 0.5; *U*_0_ (mL) is dead volume of the column (liquid volume in the column between the bottom edge of the resin bed and the outlet of the column, under process conditions *U*_0_ = 2 mL); *V_v_* (mL) is free volume in the bed (approx. 0.4 bed volume); and *m* is dry resin mass in the column (g).

**Figure 7:**
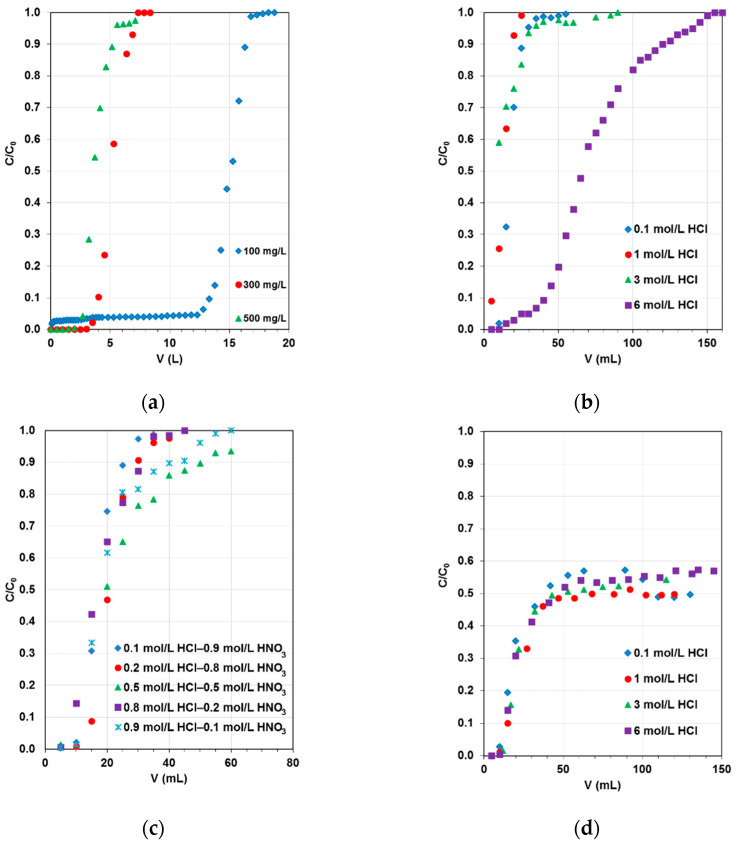
Breakthrough curves for the dye and selected heavy metal ions adsorption on S5528 resin from the systems: (**a**) 100–500 mg/L AG16, (**b**) 100 mg/L Cu(II)–0.1–6 mol/L HCl, (**c**) 100 mg/L Cu(II) –0.1–0.9 mol/L HCl–0.9–0.1 mol/L HNO_3_, (**d**) 100 mg/L Ni(II)–0.1–6 mol/L HCl.

The steep and symmetric S-shaped breakthrough curves were obtained in AG16-S5528 and Cu(II)-S5528 systems, as presented in Figure 7a–c.

For the adsorption of AG16 dye from the 100 mg/L solution, a continuous column breakthrough up to a leakage volume of 12.5 L was recorded. Furthermore, an unusual course of the breakthrough curves was observed for the adsorption of 100 mg/L Ni(II) (Figure 7d) and 100 mg/L Zn(II) from both hydrochloric acid solutions (0.1–6 mol/L HCl) and a mixture of hydrochloric and nitric acids (0.1–0.9 mol/L HCl–0.9–0.1 mol/L HNO_3_). The column parameters were calculated and are placed in Table 4.

The working capacities for AG16 were dependent on the dye concentration in the feeding solutions and decreased with increasing AG16 concentration. The values of *D_w_* and *D_b_* were in the range 2080–8380 mL/g and 374.4–1499.4, respectively.

Adsorption of Cu(II) on S5528 in the column system was practically independent of hydrochloric and nitric acid concentrations and equaled to 50 mg/L; the exception was found in 6 mol/L HCl solutions. Similar observations were found during Ni(II) adsorption of 100 mg/L Ni(II)–0.1–6 mol/L and 100 mg/L Ni(II)–0.1–0.9 mol/L HCl–0.9–0.1 mol/L HNO_3_, and *C_w_* was calculated as 50 mg/L. The working capacities for Zn(II) in 0.1 mol/L and 1 mol/L HCl were 200 mg/L, while in 3 mol/L and 6 mol/L solutions they were found to be 2000 mg/L and 1400 mg/L, respectively. In HCl (increasing up to 0.5 mol/L) and HNO_3_ (decreasing up to 0.5 mol/L) medium, the values of *C_w_* were equaled to 200 mg/L. In the 0.8 mol/L HCl–0.2 mol/L HNO_3_ and 0.9 mol/L HCl–0.1 mol/L HNO_3_ systems, the working capacities for Zn(II) were calculated to be 400 mg/L and 600 mg/L, respectively.

## 3. Materials and Methods

### 3.1. Adsorbent and Adsorbates

**Figure 8:**
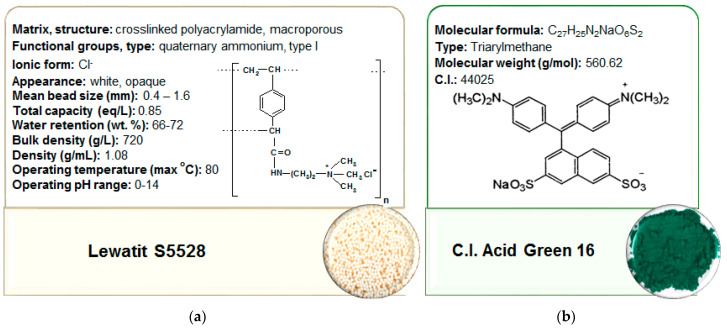
Properties of (**a**) applied anion exchange resin and (**b**) dye.

The adsorbent selected for the study was anion exchange resin Lewatit S5528 (LANXESS Deutschland GmbH, Germany). It is a macroporous, strongly basic anion exchange resin of the quaternary ammonium functional groups (type I) based on a cross-linked polyacrylate, supplied in the Cl^−^ form. Physical and chemical properties of the resin are presented in Figure 8a.

Triarylmethane type dye, i.e., C.I. Acid Green 16, was used as adsorbate. It was purchased from Boruta-Zachem (Zgierz, Poland). The physicochemical properties of the dye are presented in Figure 8b. It is used for the dyeing of wool and silk as well as printing. The stock dye solution of the initial concentration *C*_0_ = 10,000 mg/L was prepared in distilled water and the working solutions of the defined concentrations were obtained by diluting using volumetric flasks.

Besides the dye, phenol and heavy metal ions such as Zn(II), Cu(II) and Ni(II) were also adsorbents. Stock solutions containing phenol or heavy metal ions were prepared from solid phenols ZnCl_2_, CuCl_2_·2H_2_O, NiCl_2_·6H_2_O by dissolving them in ultrapure water (phenol) or HCl or a mixture of HCl and HNO_3_ (heavy metals) to maintain the desired acid concentration and ultrapure water. The above-mentioned chemicals were of analytical grade, and they were purchased from POCh S.A. (Gliwice, Poland).

Other chemical reagents such as CH_3_OH, Na_2_SO_4_, NaCl, NaOH were obtained from Avantor Performance Materials Poland S.A. (Gliwice, Poland). Purified water came from Millipore (UMCS, Poland). The surfactants, i.e., sodium dodecyl sulfate (SDS) and 2-[4-(2,4,4-trimethylpentan-2-yl)phenoxy]ethanol (Triton X100) were bought from Sigma-Aldrich (Darmstadt, Germany).

### 3.2. Adsorption Tests

In the batch adsorption method, the influence of such parameters as adsorbate concentrations, phase contact time, auxiliary presence (electrolytes: NaCl and Na_2_SO_4_; surfactants: SDS, Triton X100) were investigated as factors governing the dye, heavy metals and phenol uptake from single-component solutions. The adsorption tests were performed at 298 K using a laboratory shaker Elpin 358+ (Lubawa, Poland) at rotary *r* = 180 cpm (rotation cycles per minute), amplitude *A* = 8. The anion exchanger was separated from the solution by decantation. Then, the solution was analyzed spectrophotometrically (Cary 60 Agilent, Santa Clara, CA, USA) to determine the dye and phenol content, or by using the ASA absorption spectrometer Varian AA240FS with SIPS autosampler (Varian, Australia) to determine heavy metal ion content after the sorption process at the maximum absorbance wavelengths. The adsorption experiments were performed in triplicate with reproducibility ± 3%. The conditions for the adsorption and regeneration experiments are summarized as follows:

- The equilibrium studies: *C*_0_ = 1000–9000 mg/L AG16, *C*_0_ = 5–500 mg/L phenol, *C*_0_ = 5–500 mg/L, Cu(II)–6 mol/L HCl, *C*_0_ = 5–4000 mg/L Zn(II)–3 mol/L HCl, *C*_0_ = 5–2000 mg/L Ni(II)–0.1 mol/L HCl, *m* = 0.5 g, *V* = 50 mL, *r* = 180 cpm, *A* = 8, T = 298 K, *t* = 24 h;
- The kinetic studies: *t* = 1–240 min, *C*_0_ = 100, 300, 500 mg/L AG16, *C*_0_ = 1, 5, 10, 50 mg/L phenol, C_0_ = 10, 50, 100 mg/L M(II)–0.1, 1, 3, 6 mol/L HCl, C_0_ = 10, 50, 100 mg/L M(II)–0.1–0.9 mol/L HCl–0.9–0.1 mol/L HNO_3_ (i.e., 0.1 mol/L HCl–0.9 mol/L HNO_3_, 0.2 mol/L HCl–0.8 mol/L HNO_3_, 0.5 mol/L HCl–0.5 mol/L HNO_3_, 0.8 mol/L HCl–0.2 mol/L HNO_3_, 0.9 mol/L HCl–0.1 mol/L HNO_3_), *m* = 0.5 g, *V*=50 mL, *r* = 180 cpm, *A* = 8, *T* = 298 K;
- Effect of auxiliaries: *C*_0_ = 500 mg/L AG16–0.1, 0.25, 0.5 g/L SDS or Triton X100; *C*_0_ = 100 mg/L M(II)–0.1, 0.25, 0.5 g/L SDS or Triton X100; *C*_0_ = 500 mg/L AG16–5, 15, 25 g/L NaCl or Na_2_SO_4_, *C*_0_ = 100 mg/L M(II)–5, 15, 25 g/L NaCl or Na_2_SO_4_, *m* = 0.5 g, *V* = 50 mL, *r* = 180 cpm, *A* = 8, T = 298 K, t = 15 min;
- Regeneration studies involved three cycles of sorption and desorption. Sorption conditions: *C*_0_ = 500 mg/L AG16, *C*_0_ = 100 mg/L, Cu(II)–6 mol/L HCl, *C*_0_ = 100 mg/L Zn(II)–3 mol/L HCl, *C*_0_ = 100 mg/L Ni(II) –0.1 mol/L HCl, *m* = 0.5 g, *V* = 50 mL, *r* = 180 cpm, *A* = 8, T = 298 K, *t* = 4 h; desorption conditions: *C*_0_ = 0.1, 1, 2 mol/L HCl, HNO_3_, H_2_SO_4_, NaCl or NaOH regenerants for M(II) removal, *C*_0_ = 1 mol/L HCl, 1 mol/L HCl + 50% *v*/*v* MeOH, 1 mol/L NaCl, 1 mol/L NaCl + 50% *v*/*v* MeOH, 1 mol/L NaOH and 1 mol/L NaOH + 50% *v*/*v* MeOH regenerants for AG16 removal, *m* = 0.5 g, *V* = 50 mL, *r* = 180 cpm, *A* = 8, T = 298 K, *t* = 4 h.

In the dynamic method, a set of ion exchange columns (Ø = 1 cm) was used in which 10 mL of the swollen resin was placed. A dye or heavy metal ion solution of the specified concentration was let into the bed at the rate of 0.4 cm^3^/min. The concentration of dye and heavy metal ions in the effluent was determined as described above. Composition of the influent: *C*_0_ = 100, 300, 500 mg/L AG16, 100 mg/L Cu(II) or Zn(II) or Ni(II)– 0.1, 1, 3, 6 mol/L HCl, *C*_0_ = 100 mg/L Cu(II) or Zn(II) or Ni(II)–0.1–0.9 mol/L HCl–0.9–0.1 mol/L HNO_3_ (i.e., 0.1 mol/L HCl–0.9 mol/L HNO_3_, 0.2 mol/L HCl–0.8 mol/L HNO_3_, 0.5 mol/L HCl–0.5 mol/L HNO_3_, 0.8 mol/L HCl–0.2 mol/L HNO_3_, 0.9 mol/L HCl–0.1 mol/L HNO_3_).

## 4. Conclusions

C.I. Acid Green 16, heavy metal ions (zinc(II), nickel(II), copper(II)) and phenol adsorption processes of the strongly basic anion exchanger with quaternary ammonium functionalities and a polyacrylamide matrix were investigated. Based on the Langmuir isotherm model, the macroporous anion exchanger Lewatit S5528 showed monolayer adsorption capacities as 538 mg/g, 14.5 mg/g and 5.8 mg/g for AG16, phenol and Cu(II), respectively. The Freundlich model seemed to be the better option for the description of the equilibrium sorption data of Zn(II) (*k_F_* = 0.179 mg^1−1/n^ L^1/n^/g) and Ni(II) (*k_F_* = 0.048 mg^1−1/n^ L^1/n^/g) on S5528. Kinetic data indicated that the amount of contaminants adsorbed on S5528 was dependent on the initial concentration and the phase contact time. The pseudo-second-order kinetic model fitted experimental data better than pseudo-first-order or intraparticle diffusion models. The effect of salt (NaCl, Na_2_SO_4_) and surfactant (SDS, Triton X100) concentrations influenced the resin performance. Regeneration studies performed in three cycles of sorption and desorption revealed that the uptake of heavy metal ions and dyes did not change significantly, while the desorption yield of AG16 was effective using 1 mol/L HCl + 50% *v*/*v* MeOH and 1 mol/L NaCl + 50% *v*/*v* MeOH solutions. Column experiments confirmed the preferential applicability of S5528 in AG16 and Zn(II) removal, rather than Cu(II) or Ni(II). The conducted research is of great cognitive importance, especially in the design of modern technological solutions used in the treatment of wastewater containing toxic substances of various types.

## Figures and Tables

**Table 1:** Values of parameters of the Langmuir, Freundlich and Temkin isotherms calculated for AG16, phenol and Cu(II) sorption on S5528 anion exchange resin.

Model	Parameters	AG16	Phenol	Cu(II)
Langmuir-L	*Q*_0_ (mg/g)	538	14.5	5.8
	*k_L_* (L/mg)	0.007	0.057	0.003
	*R* ^2^	0.997	0.989	0.586
Langmuir-NL	*Q*_0_ (mg/g)	458	14.3	8.3
	*k_L_* (L/mg)	0.016	0.046	0.002
	*MPSD*	0.256	3.9	1.3
	*R* ^2^	0.914	0.949	0.901
	$R_{adj}^{2}$	0.897	0.943	0.889
Freundlich-L	$k_{F}$ (mg^1−1/n^ L^1/n^/g)	64.5	1.07	0.021
	1/*n*	0.257	0.504	0.905
	*R* ^2^	0.928	0.780	0.927
Freundlich-NL	$k_{F}$ (mg^1−1/n^ L^1/n^/g)	58.9	0.369	0.018
	1/*n*	0.285	0.663	0.909
	*MPSD*	0.256	6.3	2.0
	*R* ^2^	0.949	0.861	0.837
	$R_{adj}^{2}$	0.939	0.844	0.818
Temkin-L	*b_T_* (J g/mol mg)	31.8	1263	3167
	*A* (L/mg)	0.28	3.13	0.096
	*R* ^2^	0.984	0.978	0.913
Temkin-NL	*b_T_* (J g/mol mg)	315	694	694
	*A* (L/mg)	0.25	4.45	4.45
	*MPSD*	0.102	72.5	585
	*R* ^2^	0.986	0.978	0.900
	$\;R_{adj}^{2}$	0.981	0.975	0.888

**Table 2:** Comparison of the sorption properties of various adsorbents for AG16, phenol and heavy metal ions uptake based on a literature review.

Adsorbent	Sorption Capacity and Experimental Conditions	Ref.
**AG16**		
Magnetic geopolymer	*q_e_* = 108 mg/g, * T = 298K, ** a.d. = 0.75 g/L	[59]
Rice bran-based activated carbon	*q_e_* = 1.05–1.36 mg/g, T = 293–328 K, a.d. = 1 g/50 mL	[60]
Lignite	*q_e_* = 19.32 mg/g, room temperature, a.d. = 1 g/50 mL	[61]
Strongly basic anion exchange resin (polystyrene matrix, Lewatit S6368A)	*q_e_* = 625–811 mg/g, T = 293–333 K, a.d. = 0.5 g/50 mL	[62]
Strongly basic anion exchange resin (polyacrylate matrix, Lewatit S5528)	*q_e_* = 538 mg/g, T = 298 K, a.d. = 0.5 g/50 mL	This study
**Phenol**		
Ultrasound-assisted sulphuric acid-treated pea shells	*q_e_* = 122–157 mg/g, T = 293–318 K, a.d. = 0.1 g/100 mL	[63]
Modified bentonite	*q_e_* = 3–10 mg/g, T = 298 K, a.d. = 0.5 g/30 mL	[64]
Polystyrene non-functionalized resin (Amberlite XAD 4)	*q_e_* = 75–78 mg/g, T = 293–333 K, a.d. = 0.05 g/50 mL	[65]
Weakly basic anion exchange resin (polystyrene matrix, Amberlite IRA96C)	*q_e_* = 47–56 mg/g, T = 293–333 K, a.d. = 0.05 g/50 mL	
Aminated polymeric resin NDA103 (polystyrene matrix)	*q_e_* = 122–131 mg/g, T = 293–333 K, a.d. = 0.05 g/50 mL	
Strongly basic anion exchange resin (polyacrylate matrix, Lewatit S5528)	*q_exp_* = 14.5 mg/g, T = 298 K, a.d. = 0.5 g/50 mL	This study
**Heavy metal ions**		
Silica-alumina-based adsorbent	*q_e_* = 0.123 mg Cu(II)/g, *q_e_* = 0.198 mg Ni(II)/g, *q_e_* = 4.16 mg Zn(II)/g, T = 293 K, a.d. = 0.05 g/50 mL	[66]
Carbonaceous adsorbents prepared from spent ion exchange resins	*q_e_* = 6.38–6.96 mg Cu(II)/g, T = 295 K, a.d. = 0.1 g/20 mL	[67]
Strongly basic anion exchange resin (polystyrene matrix, Amberlite IRA402)	*q_e_* = 56.67 mg Cu(II)/g, *q_e_* = 38.39 mg Zn(II)/g, T = 295 K, a.d. = 0.5 g/50 mL	[47]
Strongly basic anion exchange resin (polyacrylic matrix, Amberlite IRA458)	*q_e_* = 51.1 mg Cu(II)/g, *q_e_* = 43.09 mg Zn(II)/g, T = 295 K, a.d. = 0.5 g/50 mL	
Strongly basic anion exchange resin (polyacrylate matrix, Lewatit S5528)	*q_e_* = 0.048 mg^1−1/n^ L^1/n^ Ni(II)/g, *q_e_* = 0.179 mg^1−1/n^ L^1/n^Zn(II)/g, *q_e_* = 8.3 mg Cu(II)/g, T = 298 K, a.d. = 0.5 g/50 mL	This study

* T—temperature; ** a.d.—adsorbent dose.

**Table 3:** Kinetic parameters for AG16, Cu(II) and Zn(II) sorption on Lewatit S5528.

Parameters		AG16			Cu(II) *	Zn(II) *
***q_e,exp_* (mg/g)**		**100 mg/L**	**300 mg/L**	**500 mg/L**	**100 mg/L**	**100 mg/L**
		9.9	29.9	49.7	1.5	7.5
**PFO-L**	*q_e_* (mg/g)	0.2	0.7	2	0.02	0.6
	*k_1_* (1/min)	0.013	0.014	0.019	0.035	0.062
	*R* ^2^	0.239	0.290	0.435	0.524	0.788
**PFO-NL**	*q_e_* (mg/g)	1.5	7.6	7.2	1.5	7.2
	*k_1_* (1/min)	1.3	1.9	2.1	1.3	2.1
	*R* ^2^	0.934	0.961	0.829	0.953	0.829
	$R_{adj}^{2}$	0.915	0.949	0.780	0.940	0.780
	*MPSD*	0.0071	0.0078	5.1	0.0032	0.0226
**PSO-L**	*q_e_* (mg/g)	9.9	29.8	49.8	1.5	7.5
	*k*_2_ (g/mg min)	0.738	0.135	0.040	4.7	0.317
	*R* ^2^	1.0	1.0	1.0	1.0	1.0
	*h*	72.8	119.9	99.1	10.1	17.9
**PSO-NL**	*q_e_* (mg/g)	10.1	30.6	51.9	1.5	7.4
	*k*_2_ (g/mg min)	0.218	0.046	0.014	1.9	0.654
	*R* ^2^	0.973	0.969	0.937	0.966	0.766
	$R_{adj}^{2}$	0.968	0.965	0.929	0.956	0.699
	*MPSD*	0.0026	0.0061	0.0309	0.0023	0.0092
**IPD**	*q_e_* (mg/g)	27.1	76.9	157	2.9	10.7
	*k_i_* (mg/g min^0.5^)	1.4	3.9	8.9	0.1	0.3
	*R* ^2^	0.829	0.763	0.765	0.676	0.977
	$R_{adj}^{2}$	0.488	0.526	0.531	0.351	0.954

* in 6 mol/L HCl.

**Table 4:** Column parameters calculated for AG16 and Cu(II) sorption on S5528.

System	*C_w_* (mg/L)	*D_w_* (mL/g)	*D_b_*
**AG16**			
100 mg/L AG16	118	8330	1499
300 mg/L AG16	90	2774	499
500 mg/L AG16	75	2080	374
**Cu(II)**			
100 mg/L Cu(II)–0.1 mol/L HCl	50	1.1	0.2
100 mg/L Cu(II)–1 mol/L HCl	50	3.9	0.7
100 mg/L Cu(II)–3 mol/L HCl	50	6.1	1.1
100 mg/L Cu(II)–6 mol/L HCl	100	33.9	6.1
100 mg/L Cu(II)–0.1 mol/L HCl–0.9 mol/L HNO_3_	50	6.7	1.2
100 mg/L Cu(II)–0.2 mol/L HCl–0.8 mol/L HNO_3_	50	7.8	1.4
100 mg/L Cu(II)–0.5 mol/L HCl–0.5 mol/L HNO_3_	50	7.8	1.4
100 mg/L Cu(II)–0.8 mol/L HCl–0.2 mol/L HNO_3_	50	7.8	1.4
100 mg/L Cu(II)–0.9 mol/L HCl–0.1 mol/L HNO_3_	50	7.8	1.4

## Data Availability

All data used to support the findings of this study are included within the article.

## Sample Availability

Samples of the compounds are not available from the authors.
